# Quantifying training load and intensity in elite male ice hockey according to game-related contextual variables

**DOI:** 10.5114/biolsport.2023.114282

**Published:** 2022-05-20

**Authors:** Vincenzo Rago, Magni Mohr, Jeppe F. Vigh-Larsen

**Affiliations:** 1 Faculty of Health and Sports Sciences, Universidade Europeia, Lisbon, Portugal; 2 Department of Sports Science and Clinical Biomechanics, Faculty of Health Sciences, University of Southern Denmark, Odense, Denmark; 3 Centre of Health Sciences, Faculty of Health, University of the Faroe Islands, Tórshavn, Faroe Islands; 4 Research Unit for Exercise Biology, Department of Public Health, Aarhus University, Aarhus, Denmark

**Keywords:** Team sports, Physiology, Time motion, Heart rate, Wearable technology

## Abstract

We aimed to quantify training load (TL) and intensity during practice sessions according to game-related contextual variables (game outcome, opponent standard, game location) in an elite male ice hockey team. Practice data were collected using a wearable 200-Hz accelerometer, heart rate (HR) recording, and session-rating of perceived exertion (s-RPE) throughout 23 sessions (n = 306 files). The reference team performed a greater number of accelerations, decelerations, spent longer time > 85% maximum HR (t85%HR_max_) and reported greater s-RPE after losing a game compared to a win (r = 0.13–0.19). Moreover, a lower number of accelerations, decelerations, t85%HR_max_ and s-RPE (r = 0.15–0.45) were found before playing against a top-ranked opponent. In contrast, more accelerations, decelerations, longer t85%HR_max_ and greater s-RPE were observed after playing against a top-ranked team opponent (r = 0.15–0.41). The players performed more accelerations/min, spent more t85%HR_max_ and reported greater s-RPE before playing an away game (r = 0.13–0.22). Weekly TL seems to slightly increase after losing a game, when preparing a game against a weaker opponent, after playing against a stronger opponent, and when preparing an away game. On the other hand, training intensity seems not to be affected by game-related contextual variables. Thus, ice hockey practitioners involved with TL monitoring should consider the interplay of the numerous variables that influence the volume of prescribed training and the actual training responses in each individual player.

## INTRODUCTION

Training load (TL) monitoring, which has become a key area of attention of sports science support in contemporary team sports, aims to generate information concerning attainment of peak performance, to enable a certain level of protection against injury, and to provide an evidence-based and systematic approach to management of decisions when prescribing training [1]. In ice hockey, this is usually done using wearable technology embedding accelerometer and heart rate (HR) or collecting the rating of perceived exertion (RPE) during practice sessions and official games. However, limited attempts have been done to describe TL measures and intensity in elite ice hockey [2–5].

The external TL (ETL) describes the amount of physical work completed by the athlete. The ETL has recently been described in elite male [3, 2, 5] and female [4] ice hockey players using wearable technology. Beyond the relevant information available from ETL, different athletes may produce the same exercise output while experiencing different physiological and/or perceptual loading. In this context, the internal TL (ITL) is the individual athlete’ response induced by the ETL stimulus. ITL in ice hockey is usually determined using HR- and RPE-based parameters [2]. Recent findings have shown that the official game was the most demanding weekly session, and that day-to-day variations were more pronounced in HR- and RPE-based parameters rather than accelerations and decelerations [6]. Intense skating efforts performed during practice seem to be associated with that of competition, but not physiological and perceptual demands [6]. However, to our knowledge, no information has been reported regarding the influence of game-related variables (game outcome, opponent standard and game location) on TL and intensity in ice hockey.

Previous observational studies revealed that TL and intensity during the week can be affected by previous or upcoming game-related contextual factors in other team sports such as soccer [7–11], rugby union [12, 13] and basketball [14] showing controversial findings. For example, lower session-RPE (s-RPE; RPE × duration) has been reported after winning a game in U19 soccer [15] and semi-professional basketball players [14], or after losing a game in professional rugby union [12]. On the other hand, greater total distance covered was found in the week after winning a game in elite soccer players [7]. Additionally, soccer players showed greater high-speed running (> 16 km · h^-1^) when preparing a game against a weaker opponent (bottom five of the league rank [7]), whereas basketball players perceived greater s-RPE before facing a stronger opponent (first five of the league rank [14]). Furthermore, various team-sports players experienced different [15] or similar [7, 13, 11] TL when preparing an away or a home game. However, this information is yet to be explored in ice hockey. Therefore, the aim of this study was to quantify TL and intensity during practice sessions in elite Danish male ice hockey according to game-related contextual variables.

## MATERIALS AND METHODS

### Participants

17 elite Danish male ice hockey players (6 defensemen and 11 forwards; Mean ± standard deviation; age = 26 ± 5 yrs old, height = 181 ± 6 cm, body mass = 81.6 ± 6.9 kg, body fat = 13.6 ± 2.2% and HR_max_ = 193 ± 7 bpm) competing at the highest Danish national level of play were regularly monitored in the context of their team practice sessions and game routines. Written informed consent was obtained from all the subjects. The Institutional Review Board of the University Southern Denmark approved the study, and written informed consent was obtained from all participants. The study adhered to the ethical code of the Declaration of Helsinki, and procedures were in line with established ethical standards in sports sciences [16].

### Design

Practice sessions and competitive game data were collected using a wearable 200-Hz accelerometric system, HR and RPE throughout a four-week (29 days) competitive period, between February and March 2020. This was at the final stages of the regular season before a short break leading into the playoffs. Individual reconditioning sessions for injured players were excluded for analysis. Players participated in 23 practice sessions and 8 competitive games. Practice data included six sessions (92 files) on game-day minus 2, eight sessions (118 files) on game-day minus 1, and seven sessions (96 files) on game-day minus 0. Game-day minus 4 and 3 included one session (16 files) and as such were excluded from the study. The final sample comprised 306 practice sessions (median [range] = 18.5 [9–21] per player). To remove the effect of training frequency, TL and intensity data were expressed as average between game-day minus sessions as previously described [7]. The team won 4 games and loss 4 games and played 4 home and 4 away games. The opponent standard was classified as top (current 1^st^ to 3^rd^ of the rank), mid (4^th^ to 6^th^ team) and bottom (7^th^ to 8^th^). During games, only players who spent a minimum of 4 min on the ice were considered [2]. No input was given about the research design to the technical staff throughout the data collection period. Data were collected in the context of the regular player’ monitoring routine.

### Procedures

The session-duration was regularly quantified by team’ strength and conditioning coach. Acceleration and HR data were recorded using a wearable device incorporating a 200-Hz accelerometer and gyroscope (Polar Team Pro system, Polar, Kempele, Finland) and a 1-s interval telemetric system, respectively. The device (weight: 39 g; dimension: 36 mm × 68 × 13 mm) was placed on the lower sternum using an elastic band. Data were stored in the device and downloaded using the manufacturing software (POLAR Team Pro, Software version 1.3.1, POLAR, Polar Electro Oy, Kempele, Finland). Acceleration variables included the number of total accelerations (Acc_tot_), total decelerations (Dec_tot_), which both were expressed either in absolute values (number of efforts) and scaled by individual exposure time (efforts per min). The accelerometric system was tested for reliability by the study authors before the commencement of the study, and it provided comparable results to similar systems currently used in ice hockey (coefficient of variation ranging from 0.54 to 8.6%; [17]).

The HR indicators included time > 85% HR_max_ (t85HR_max_) and mean HR (HR_mean_), based on previous ice hockey studies [18, 2]. The players’ HR_max_ was obtained in a previously described ice hockey specific incremental test [19].

The same strength and conditioning coach collected the player’s individual RPE using Borg’s category ratio scale after training sessions. Player RPE was collected in isolation to avoid the potential effects of peer pressure 15–30 min after each training session, ensuring that the perceived effort reflected the whole session and not the most recent exercise intensity [20, 21]. The overall TL was described by the session-RPE, which was calculated by multiplying the RPE score (0 to 10 AU) by the individual session’ duration (in min, excluding games’ warm-up) [22]. The validity of the s-RPE in ice hockey has been recently reported in relation to objective TL indicators (*r* = 0.70–0.89) [2].

### Statistical analysis

Differences between game-related contextual factors were analyzed using a linear mixed model with unstructured covariance, taking into consideration the fact that the participants differed in respect of the number of training sessions in which they participated [23]. The specific contextual factor was set as fixed effect, individual subjects were set as random effects, and TL was the dependent variable. When a significant effect was found, pairwise comparisons were analyzed using a post-hoc Bonferroni test. The t statistic derived from the mixed model and from the paired sample’ t test was converted to effect sizes’ correlations [24] and qualitatively interpreted as: trivial (*r* ≤ 0.1), small (*r* = 0.1–0.3), moderate (*r* = 0.3–0.5), large (*r* = 0.5–0.7), very large (*r* = 0.7–0.9) and almost perfect (*r* ≥ 0.9) [25].

Descriptive statistics were presented as estimated marginal mean ± standard error. Statistical significance was set at *P* ≤ 0.05. Data analyses were performed using Statistical Package for Social Science software (IBM SPSS Statistics for Windows, Version 26.0. Armonk, NY).

## RESULTS

### Playing position

Defensemen and forwards were exposed to similar training duration (*P* = 0.589). However, Defensemen were imposed slightly to moderately greater acceleration- and HR-based TL and intensity than forwards (*r* = 0.23–0.38; *P* < 0.05). No between-position differences were observed in s-RPE (*P* = 0.867). A detailed description of average TL and intensity in an elite Danish Ice Hockey team according to playing position is reported in Table 1.

**TABLE 1:** Average training load and intensity in an elite Danish Ice Hockey team according to playing position (*n* = 306 files).

Variable	Defensemen	Forwards	r(95 %CI)	*P*
Duration (min)	53.1 ± 1.6	54.2 ± 1.2	0.03 (-0.09; 0.15)	0.589
Acc_tot_.(count)	309.0 ± 9.1	264.1 ± 6.8	0.23 (0.12; 0.34)	< 0.001
Dec_tot_. (count)	307.2 ± 9.1	264.2 ± 6.8	0.22 (0.11; 0.33)	< 0.001
Acc_tot_./ min (count)	5.90 ± 0.08	4.86 ± 0.10	0.52 (0.43; 0.6)	< 0.001
Dec_tot_./ min (count)	5.86 ± 0.08	4.86 ± 0.10	0.52 (0.42; 0.6)	< 0.001
Time > 85%HR_max_ (min)	12.1 ± 0.7	6.1 ± 0.5	0.38 (0.27; 0.47)	< 0.001
HR_mean_(%HR_max_)	73.7 ± 0.6	67.0 ± 0.4	0.49 (0.40; 0.58)	< 0.001
s-RPE (AU)	226.8 ± 12.7	224.1 ± 10.3	0.01 (-0.13; 0.15)	0.867

Acc_tot_, total accelerations; AU, arbitrary units; CI, confidence intervals; Dec_tot_, total decelerations; HR_max_, maximum heart rate; s-RPE, session-rating of perceived exertion.

### Game outcome

No significant differences in TL and intensity were observed before a loss compared to a win (*P* > 0.05). However, players had slightly longer session-duration, performed a greater number of Acc_tot_, Dec_tot_, spent more t85%HR_max_ and reported greater s-RPE, after losing a game compared to a win (*r* = 0.13–0.19; *P* < 0.05). No significant differences were observed in training intensity after losing or winning a game (Acc_tot_/min, Dec_tot_/min, HR_mean_; *P* > 0.05). A detailed description of average TL and intensity in an elite Danish Ice Hockey team according to game outcome is reported in Table 2.

**TABLE 2:** Average training load and intensity in an elite Danish Ice Hockey team according to game outcome (*n* = 306 files).

Variable	Before				After			
	Loss	Win	*r*(95 %CI)	*P*	Loss	Win	r(95 %CI)	*P*
Duration (min)	52.9 ± 1.4	54.5 ± 1.3	0.05 (-0.07; 0.17)	0.418	56.3 ± 1.5	52.2 ± 1.2	0.13 (0.01; 0.24)	0.033
Acc_tot_.(count)	278.1 ± 8.5	281.7 ± 7.4	0.02 (-0.10; 0.14)	0.746	300.0 ± 8.7	266.6 ± 7.1	0.18 (0.06; 0.29)	0.003
Dec_tot_. (count)	278.1 ± 8.5	280.7 ± 7.4	0.01 (-0.11; 0.13)	0.823	299.5 ± 8.6	266 ± 7.1	0.18 (0.06; 0.29)	0.003
Acc_tot_./ min (count)	5.23 ± 0.09	5.24 ± 0.08	0.01 (-0.11; 0.13)	0.921	5.33 ± 0.09	5.17 ± 0.08	0.08 (-0.04; 0.20)	0.166
Dec_tot_./ min (count)	5.23 ± 0.09	5.20 ± 0.08	0.01 (-0.11; 0.13)	0.838	5.32 ± 0.09	5.15 ± 0.07	0.09 (-0.03; 0.21)	0.135
Time > 85%HR_max_ (min)	9.0 ± 0.7	7.7 ± 0.6	0.08 (-0.04; 0.20)	0.187	9.70 ± 0.70	7.3 ± 0.6	0.16 (0.04; 0.27)	0.009
HR_mean_(%HR_max_)	70.3 ± 0.6	68.8 ± 0.5	0.11 (-0.01; 0.23)	0.058	70.3 ± 0.6	68.8 ± 0.5	0.11 (-0.01; 0.23)	0.062
s-RPE (AU)	235.9 ± 12.8	218.4 ± 10.1	0.08 (-0.06; 0.21)	0.287	250.7 ± 12.1	207.0 ± 10.2	0.19 (0.05; 0.32)	0.006

Acc_tot_, total accelerations; AU, arbitrary units; CI, confidence intervals; Dec_tot_, total decelerations; HR_max_, maximum heart rate; s-RPE, session-rating of perceived exertion.

### Opponent standard

Players were exposed to moderately lower training duration, performed less amount of Acc_tot_, Dec_tot_ (*r* = 0.37–0.45; *P* < 0.01) and spent slightly lower t85%HR_max_ and reported lower s-RPE (*r* = 0.15–0.28; *P* < 0.05) before playing against a top-ranked opponent compared to mid- and bottom-ranked opponents. On the other hand, players were exposed to greater training duration, performed more Acc_tot_, Dec_tot_ (*r* = 0.18–0.41; *P* < 0.01) and spent greater t85%HR_max_ (*r* = 0.15–0.32; *P* < 0.05) after playing against a top-ranked team compared to mid- and bottom-ranked opponents. Similarly, players reported greater s-RPE after playing against a top-ranked team compared to a mid-ranked team *r* = 0.21 [0.07–0.34]; *P* = 0.002). No significant differences were observed in training intensity according to the opponent standard (Acc_tot_/min, Dec_tot_/min, HR_mean_; *P* > 0.05). A detailed description of average TL and intensity in an elite Danish Ice Hockey team according to the opponent standard is reported in Table 3.

**TABLE 3:** Average training load and intensity in an elite Danish Ice Hockey team according to the opponent standard (*n* = 306 files).

Variable	Before				After			
	Bottom	Mid	Top	Pairwise comparisons	Bottom	Mid	Top	Pairwise comparisons
Duration (min)	58.8 ± 1.4	58.8 ± 1.4	41.9 ± 1.5	Bottom, Mid > Top	54.0 ± 1.3	45.1 ± 1.6	62.2 ± 1.6	Top > Bottom > Mid
Acc_tot_.(count)	312.0 ± 8.3	300.2 ± 8.6	217.7 ± 9.3	Bottom, Mid > Top	276.4 ± 7.9	239.9 ± 10	325.8 ± 9.9	Top > Bottom > Mid
Dec_tot_. (count)	311.2 ± 8.3	300.0 ± 8.6	217.1 ± 9.2	Bottom, Mid > Top	276.5 ± 7.9	237.4 ± 9.9	326.1 ± 9.8	Top > Bottom > Mid
Acc_tot_./ min (count)	5.36 ± 0.10	5.10 ± 0.11	5.23 ± 0.11	-	5.14 ± 0.09	5.33 ± 0.11	5.28 ± 0.11	-
Dec_tot_./ min (count)	5.33 ± 0.09	5.09 ± 0.10	5.21 ± 0.10	-	5.15 ± 0.08	5.27 ± 0.11	5.27 ± 0.11	-
Time > 85%HR_max_ (min)	9.2 ± 0.8	9.1 ± 0.8	6.1 ± 0.9	Bottom, Mid > Top	8.3 ± 0.7	6.2 ± 0.9	10.3 ± 0.9	Top > Bottom > Mid
HR_mean_(%HR_max_)	69.4 ± 0.7	68.9 ± 0.7	70.1 ± 0.7	-	69.4 ± 0.6	68.7 ± 0.8	70.2 ± 0.8	-
s-RPE (AU)	236.3 ± 12.3	251.6 ± 12.6	168.7 ± 15.3	Bottom, Mid > Top	224 ± 11.3	188.9 ± 16.4	254.9 ± 14.3	Top > Mid

Acc_tot_, total accelerations; AU, arbitrary units; CI, confidence intervals; Dec_tot_, total decelerations; HR_max_, maximum heart rate; s-RPE, session-rating of perceived exertion.

### Game location

Players were exposed to a slightly greater training duration, Acc_tot_/min, t85%HR_max_ and reported greater s-RPE before an away game compared to a home game (r = 0.13–0.22; *P* < 0.05). No significant differences in TL and intensity were observed after a home or away game (*P* > 0.05). A detailed description of average TL and intensity in an elite Danish Ice Hockey team according to game location is reported in Table 4.

**TABLE 4:** Average training load and intensity in an elite Danish Ice Hockey team according to game location (n = 306 files).

Variable	Before				After			
	Away	Home	r(95 %CI)	*P*	Away	Home	r(95 %CI)	*P*
Duration (min)	57.2 ± 1.5	51.6 ± 1.2	0.18 (0.06; 0.29)	0.004	52.9 ± 1.7	54.3 ± 1.1	0.04 (-0.08; 0.16)	0.495
Acc_tot_.(count)	290.3 ± 8.9	273.6 ± 7.2	0.09 (-0.03; 0.2)	0.146	272.1 ± 10.0	283.8 ± 6.8	0.06 (-0.06; 0.18)	0.334
Dec_tot_. (count)	292.0 ± 8.9	271.6 ± 7.1	0.11 (-0.01; 0.22)	0.074	270.1 ± 9.9	283.9 ± 6.7	0.07 (-0.05; 0.19)	0.250
Acc_tot_./ min (count)	5.07 ± 0.09	5.34 ± 0.07	0.14 (0.02; 0.25)	0.025	5.20 ± 0.1	5.25 ± 0.07	0.02 (-0.1; 0.14)	0.725
Dec_tot_./ min (count)	5.08 ± 0.09	5.30 ± 0.07	0.11 (0; 0.23)	0.059	5.16 ± 0.1	5.24 ± 0.07	0.04 (-0.08; 0.15)	0.554
Time > 85%HR_max_ (min)	9.5 ± 0.7	7.4 ± 0.6	0.13 (0.01; 0.25)	0.031	7.3 ± 0.8	8.7 ± 0.6	0.08 (-0.03; 0.2)	0.163
HR_mean_(%HR_max_)	69.8 ± 0.6	69.2 ± 0.5	0.04 (-0.08; 0.16)	0.503	68.7 ± 0.7	69.8 ± 0.5	0.08 (-0.04; 0.2)	0.197
s-RPE (AU)	259.1 ± 13.2	207.2 ± 9.6	0.22 (0.08; 0.35)	0.002	217.3 ± 13.6	229.3± 9.8	0.05 (-0.09; 0.19)	0.475

Acc_tot_, total accelerations; AU, arbitrary units; CI, confidence intervals; Dec_tot_, total decelerations; HR_max_, maximum heart rate; s-RPE, session-rating of perceived exertion.

## DISCUSSION

The main finding of this study was that weekly TL in a top-class Danish male ice hockey team seems to be affected by game-related contextual variables. Like other team sports, these effects appear more evident in relation to the standard of the opponent and the results of the previous and following games rather than game location. Although greater training demands were imposed on defensemen compared to forwards, we found that players were exposed to (i) greater training volume after losing a game, (ii) lower TL when preparing a game against a stronger opponent, (iii) greater TL after playing against a stronger opponent and (iv) greater training duration, and acceleration frequency and cardiovascular loading when preparing an away game.

Our positional analysis revealed that although defensemen and forwards were exposed to similar training duration, defensemen were imposed slightly to moderately greater acceleration- and HR-based TL and intensity than forwards (*r* = 0.23–0.38). This contrasts with recent findings by Allard et al. [3] showing similar positional demands in on-ice load during practice sessions. However, our findings are similar to that observed in professional soccer, showing greater weekly ETL demands in defenders compared to forwards [9]. However, based on the correlation between speed, acceleration and HR parameters in team sports games [26], the present results are partially supported by that of Lignell et al. [27] showing greater moderate (14–17 km · h^-1^) and fast (17–21 km · h^-1^) speed skating covered by defensemen during one official game. No between-position differences were observed in s-RPE, possibly suggesting that defensemen were better able to tolerate greater physical demands than forwards or that the work pattern may have differed (e.g., forwards accumulating their efforts in shorter bursts resulting in similar strain despite less absolute load). However, previous research has revealed similar on-ice change of direction, sprint and aerobic capacity between playing positions in elite ice hockey players [28, 29]. In this context, further studies are warranted to better elucidate positional differences in practice and match requirements in relation to fitness profiles. Alternatively, forwards may have performed a lower amount of brief intense actions leading to a lower HR kinetics compared to longer duration efforts observed in defensemen at a slightly lower intensity. A limitation in the comparison of playing position in ice hockey is that the forward lines are normally better represented than defensive pairings, which is also the case for the reference team in the present study. Thus, during specific team drills time on ice may reflect the game and be higher for defensemen than forwards.

The players in the present study were exposed to slightly longer session-duration, performed a greater number of accelerations and decelerations, as well as had higher heart rate loading and s-RPE, after losing a game compared to a win (see Table 2). This is supported by recent findings in elite soccer showing higher weekly total distance covered and high-speed running after losing a match [7, 8]. Moreover, our findings are partially supported by higher weekly s-RPE reported after a loss or draw compared to a win in U19 soccer [15] and semi-professional basketball [14]. On the other hand, our findings contrast with that observed in rugby union showing reduced s-RPE after a loss [12]. Additionally, no significant differences were observed in training intensity after losing or winning a game. Thus, the reason for the higher global TL after a loss may mainly be caused by longer sessions, potentially because coaches might be under pressure, but this does not appear to affect training intensity.

The investigated team was exposed to moderately lower training duration, performed a lower number of accelerations and decelerations, as well as experienced lower cardiovascular loading and reported lower s-RPE (Table 3) before playing against a top-ranked opponent compared to mid- and bottom-ranked opponents. This is in accordance with previous findings in basketball showing lower s-RPE before facing a top-level opponent [14], but in contrast to professional soccer [7] and rugby union [13] showing unclear differences in various TL variables before facing different opponents. The reduced training volume before playing against a stronger opponent could be attributed to the greater physical demands commonly observed in team sports, when competing against stronger opponents [30]. Indeed, coaches may reduce the volume of high-intensity activity in favor of tactical preparation when preparing a game against top-level opponents simply to recover and avoid fatigue [31] while focusing on other components such as psychological preparation. This could possibly explain the higher rate of mechanical work when preparing for a game against a weaker team. Alternatively, increased work rate with stable physiological responses might indicate improved cardiorespiratory fitness and readiness to train and compete [32]. On the other hand, players were exposed to greater training duration and higher acceleration and deceleration count after playing against a top-ranked team compared to mid- and bottom-ranked opponents, which is supported by findings in professional soccer demonstrating increased total distance covered and high-speed running [7]. Additionally, cardiovascular loading and rating of perceived exertion were higher after playing against a top-ranked team, which contrasts with professional soccer [15, 7], basketball [14] and rugby union [13] observing trivial opponent effects on ITL. Although it is expected that coaches decrease the weekly TLs of starting players as a recovery strategy due to the opponent-induced match load, players might increase their work rate with a view to match selection. Collectively, it is difficult to draw conclusions about the cause-effect relationship between match-related contextual variables and changes in the volume of activity performed by the players.

In relation to game location players were exposed to a slightly greater training duration, acceleration frequency, cardiovascular taxation and s-RPE prior to an away game compared to a home game (r = 0.13–0.22). This contrasts with previous findings in soccer showing lower amount of high intensity accelerations in the week before an away game [7]. On the other hand, no significant differences in TL and intensity were observed after a home or away game, which is supported by previous findings in professional soccer [7], basketball [14] and rugby union [13]. This could result from players displaying residual fatigue after away games. Consequently, training intensity could have been reduced while maintaining training intensity because of cumulative loads over the previous days. On the other hand, given that sports teams normally alternate home and away matches, the volume of high-intensity activity was higher when preparing for an away game compared to a home game. This phenomenon could perhaps be explained by the possibility of improving recovery strategy (e.g., no travel) after home games and consequently increasing the weekly training stimulus.

It is important to note various limitations inherent in the present research, with the most important concerning the impossibility of obtaining a cause-effect relationship from observational studies. First, given the reduced sample size (e.g., team study) we have not accounted for the starting status. For instance, if the weekly TL was reduced for starting players, non-starting players might have experienced higher weekly TLs than advised. Future studies are therefore warranted to address the aforementioned concerns, taking into consideration that variations in TL are therefore likely to be, firstly, a direct function of coaching decisions and, secondly, of the actual short-term contextual variables of previous and following game [15].

In summary, this is the first study to quantify the weekly TL according to different game-related contextual variables using objective indicators derived from wearable technology embedding accelerometer and HR, as well a subjective indicator (s-RPE) in an elite ice hockey team. Our findings complement previous research investigating the effect of game-related contextual variables on TL and intensity in other team sports such as soccer [7–11], rugby union [12, 13] and basketball [14] and could be considered with caution by practitioners involved with TL monitoring in top-level ice hockey. The observed effect of game-related contextual variables on TL and intensity in the present study was of small to moderate magnitudes, underlining the need to identify further factors influencing weekly TL in professional male ice hockey.

## CONCLUSIONS

Weekly TL seems to slightly vary (based on the magnitude of differences) according to the standard of the opponent, the location of a game, and the results of the previous game. Overall, TL increased after losing a game, when preparing a game against a weaker opponent, after playing against a stronger opponent, and when preparing an away game. On other hand, training intensity was unaffected by game-related contextual variables. Thus, when planning training sessions, ice hockey coaches need to consider the interplay between the numerous variables that influence the volume of prescribed training (ETL) and the actual training responses (ITL) in each individual player. However, given the observational nature of the present study, future research is warranted to clarify cause-effect between game-related contextual variables and weekly TL.
